# The Adaptation of Parliament's Multiple Roles to COVID-19

**DOI:** 10.1017/S0008423920000426

**Published:** 2020-05-06

**Authors:** Jonathan Malloy

**Affiliations:** Bell Chair in Canadian Parliamentary Democracy, Department of Political Science, Carleton University, 1125 Colonel By Drive, Ottawa, ON K1S 5B6

## Abstract

Legislatures are complex institutions that serve many purposes. While their overall roles vary in different political systems, they typically serve multiple functions, including representation, law making, scrutiny of government, public symbolism, and others. These inevitably overlap and sometimes collide; similarly, individual legislators must balance party, constituency, and personal factors in their decision making. Now, in a time of sudden and unexpected disruption amid the COVID-19 pandemic, the Parliament of Canada has been forced to rethink its complex activities under urgent and unexpected new conditions.

Legislatures are complex institutions that serve many purposes. While their overall roles vary in different political systems, they typically serve multiple functions, including representation, law making, scrutiny of government, public symbolism, and others. These inevitably overlap and sometimes collide; similarly, individual legislators must balance party, constituency, and personal factors in their decision making. Now, in a time of sudden and unexpected disruption amid the COVID-19 pandemic, the Parliament of Canada has been forced to rethink its complex activities under urgent and unexpected new conditions.

Given its inherently paradoxical nature, Parliament has struggled at times to adapt, sometimes with open partisan disagreements. In this analysis, I explore how adaptation thus far has revealed the complexity of Parliament's multiple purposes, emphasizing Parliament's law-making and scrutiny roles over others, such as representation. I suggest that moving to a fully “virtual” Parliament may further emphasize some roles at the expense of others.

## Parliament's Response to COVID-19

To assess Parliament and COVID-19, it is helpful to begin with a brief chronology (drawn from Thomas, 2020). Parliament resumed sitting after its winter break on March 9, just as Canada began to fully react to the COVID-19 pandemic. MPs and senators responded with all party agreement on March 12 to adjourn Parliament until April 20. This agreement also produced an omnibus motion, quickly approved by both the House and Senate, that passed the United States-Mexico-Canada Agreement (USMCA) trade agreement implementation bill and gave the government extraordinary spending powers until the end of June.

These initial provisions proved inadequate for the response needed, especially to the slowdown of the Canadian economy. Parliament was consequently recalled on March 24, with an agreement to limit the House to a small number of MPs, distributed proportionately by party standings and seated well apart, with similar provisions for the Senate. However, initial agreement on new legislation fell apart, with the opposition demanding changes to a provision that extended government tax and spending powers until the end of 2021. It took until the middle of the night for a deal to be reached, with the key legislation passed through the House of Commons at 6 a.m. and in the Senate later that day. The motion included authority for some Commons committees to meet remotely. A further and less rancorous emergency sitting took place on April 11, again to pass special emergency legislation.

New conflict arose as the previously set return date of April 20 approached. While nearly all parties supported continuing with a minimal crew of socially distanced legislators (except for Green Party leader Elizabeth May, who argued against in-person sittings entirely [Raj, 2020]), the Conservatives and the governing Liberals argued over the frequency of sittings. The Liberals reached agreement with the Bloc Quebecois and NDP to hold one sitting per week, to be supplemented in the future by additional remote sittings (Patel, 2020), but the Conservatives argued for three in-person sittings a week immediately, at least until virtual sittings were possible. The leaders argued very publicly, with Prime Minister Trudeau suggesting that Conservative obstruction was preventing any compromise at all, and would force all 338 MPs to return to Ottawa (Berthiaume, 2020). But these differences, though sharply worded, were not profound. They reflected a normal government impulse to expedite parliamentary business, while the Conservatives demanded more opportunity to scrutinize and hold the government to account (though as noted, this was not supported by the other opposition parties). Eventually a compromise was reached, which included the entire House sitting as a special committee on the pandemic, with both virtual and in-person sittings. This new committee comprising all MPs held its first remote sitting on April 28.

## Competing Dimensions of Parliament

The Parliament of Canada operates amid two competing dimensions or logics. These have been variously identified as “representative government” versus “responsible government” (Birch, 1964) or “legislative-centred” and “executive-centred” approaches to Parliament (Franks, 1987). I identify these as the dimensions of *representation* (emphasizing voices and individual MPs) and of *governance* (emphasizing decision making and accountability for decisions). Both are fundamental aspects of Parliament but often exist in tension, reflecting the underlying ambiguity and paradox of Parliament.

The permanent tension between the two dimensions is now exacerbated by an unanticipated crisis. And in a crisis of such overwhelming scope and urgency, it is perhaps unsurprising that the dimension of governance has dominated—the role of Parliament to consider government proposals and to hold government accountable. Thus the opposition arguments in Canada have overwhelmingly focused on *scrutiny* of government actions, rather than proposing their own alternatives. The all-night showdown on March 24–25 was driven by a refusal to give an enormous blank cheque to the government in its remarkable suggestion to award itself free tax and spending powers. The arguments leading up to the April 20 resumption focused on the need to scrutinize government, particularly in a time of frantic emergency spending. In both cases, the focus remained on government proposals and the scrutiny of government, not on considering alternatives from the opposition.

The trade-off to this has been the other dimension—representation. All-party agreements expedite the passage of legislation (governance/decision making), but at the expense of transparency and backbencher power (representation). Thus Conservative MP Scott Reid protested against being marginalized and told by his own party leaders not to come to Parliament Hill on March 24, arguing that under such conditions:

A new convention will have emerged: That on any occasion when a new and unexpected crisis arises, MPs may be ordered to stay home by their respective party leaders so that some kind of elite-level deal may be executed to apply their votes in their absence. The process, underway for several decades, of reducing MPs to mere banner-carriers, whose only purpose is to fill the blank space on the election ballot beside the party name, will have been completed. (Reid, 2020)

The selective nature of social distancing precautions, including restrictions on travel, has further risks for representation. A skeleton crew of parliamentarians can leave less room for diversity. Regional representation in the March 24–25 sitting was uneven, with overrepresentation of MPs from the proximate provinces of Ontario and Quebec but none from the North or Atlantic Canada other than New Brunswick (Thomas, 2020). Gender representation was also compromised: female MPs were slightly underrepresented in the March 24–25 House of Commons debates compared to their overall numbers in the House, comprising 25 per cent of all MPs present compared to the 29 per cent they comprise in the full chamber, while female senators were more seriously underrepresented, with 32 per cent of all senators present compared to the 46 per cent in the full chamber (Thomas, 2020). Gender participation and perspectives remains a concern for either in-person or future virtual sittings of Parliament. The Inter-Parliamentary Union has specifically addressed the gender implications of parliamentary responses to COVID-19, including obstacles for women MPs participating in remote sessions due to childcare responsibilities and other barriers (Inter-Parliamentary Union, 2020). Similar concerns may be anticipated for racialized, Indigenous, and other parliamentarians.

Overall, while there has been disagreement over the response of Parliament as an institution to the COVID-19 pandemic, the major arguments for both government and opposition have been driven by the logic of governance: the role of Parliament to make collective decisions and to hold the government to account for decisions. The other dimension of representation—focusing more on individual MPs and diversity of voices—has, at least for now, been largely absent from the arguments.

It remains to be seen how far Parliament can go in adapting itself to work remotely, and how this could further emphasize or even reshape roles and dimensions. Most of the issues discussed around “a virtual Parliament” are important but essentially technical in nature, including simultaneous translation and access for MPs in rural and remote areas. More abstract is the underlying question of *what* is being adapted—that is, what roles and dimensions of Parliament can be replicated remotely, which ones cannot, and whether technology will reshape things entirely.

The effects of technology on parliamentary behaviour have long been debated. In particular, the effect of the introduction of television in the House of Commons in 1977 remains contentious, though the most conclusive recent study suggests any effect on proceedings themselves was minor (Soroka et al., 2015). A virtual Parliament, however, forces complete dependence on technology, and more than ever prioritizes the formal proceedings of the chamber and committees, the “theatre” of Parliament. But much of Parliament, perhaps even the most important aspects, happens informally and behind the scenes. While private conversations can of course continue remotely, the absence of physical proximity will likely lead to further diminishing of informal and unscheduled interactions between parliamentarians, especially across party lines. We cannot easily predict effects here, but given Parliament's complex and at times paradoxical roles, it is unwise to assume a virtual Parliament will merely replicate the exact functions as the in-person version.

## Conclusion

The Canadian Parliament struggled to adapt as an institution to the COVID-19 pandemic. The key challenges were not necessarily technical but were rooted in longstanding tensions about the multiple roles and purpose of Parliament. The malleability of arguments about why legislatures needed to meet during a pandemic are illustrated by contrasting debates in Ottawa and Edmonton. In the House of Commons, it was the opposition pushing for more sittings, in order to heighten scrutiny on government. In Alberta, it was the government arguing for more sittings, with the opposition arguing against in-person sittings (Bellefontaine, 2020). Such contradictory dynamics suggest the underlying tensions and inherent paradoxes of Parliament. And regardless of jurisdiction, initial experiences under the COVID-19 pandemic suggest that the dimension of governance asserted itself even further in Canadian legislatures, at the expense of representation and individual and diverse legislative voices.

Parliament's adaptation to the COVID-19 pandemic gives us important new insights into how its ongoing institutional tensions manifest themselves and evolve in a sudden and unexpected new context. As Parliament moves forward into virtual versions, we can anticipate further pressures and trade-offs between the dimensions. This makes it more important than ever to understand the multiple and paradoxical roles of Parliament, and to evaluate the institution on that basis.
